# Bibliographical

**Published:** 1888-07

**Authors:** 


### BIBLIOGRAPHICAL.

THE PATHOLOGY, DIAGNOSIS AND TREATMENT OF THE DISEASES OF WOMEN. By GRAILY HEWITT, M. D., London, F. R. C. P. Professor of Midwifery and Diseases of Women, University College, and Obstetric Physician to the Hospital, etc., etc.

A new American, from the Fourth Revised and Enlarged London Edition. Edited with notes, additions and illustrations, by H. MARION SIMS, M. D. Three octavo volumes of over 1000 pages and 240 illustrations. New York: E. B. Treat, 771 Broadway. 1887.

These three volumes form numbers 7, 8, and 9 of the valuable series denominated Medical Classics. The author is a very clear and forcible writer, and he does not follow the beaten track pursued by others, but presents many new, original and valuable suggestions. For many years his great work has been accepted as a standard by the profession, not alone of England, but of America as well, and it has been adopted as a text-book in many medical colleges. The edition from which this is published was in great part rewritten, and the added experience and knowledge of ten years was embodied in it. Dr. Sims then gave it a thorough revision, offering many original comments and criticisms, and making many valuable additions to the text and the illustrations. It will be readily conceived, then, that its completeness and thoroughness cannot be called in question. It is, in fact, an exhaustive treatise of the subject, and with these volumes in his possession, the physician or specialist will have comparatively little need of anything further, for they form a library of themselves.

The work is especially valuable in its consideration of the predisposing causes of changes in the shape and position of the uterus, the fruitful source of the so-common ailments of the women of America. The author believes that these are seldom seen except when the general strength has been reduced by a systematic and frequently long continued practice of taking too little food. This he believes predisposes to uterine displacements, and the treatment recommended commences with a more careful attention to hygienic conditions and thorough nutrition. He reverses the theory that is too often advanced, and teaches that local ailments largely depend upon general ones. The illustrations will commend themselves to the general practitioner, from the fact that they are, as far as possible, of the exact natural size of that which they represent. The volumes are handsomely bound and will form an attractive as well as useful addition to any physician's library.

LECTURES ON DISEASES OF THE HEART. Delivered at the College of Physicians and Surgeons, New York. By ALONZO CLARK, M. D., LL. D., Emeritus Professor of the Principles and Practice of Medicine. New York: E. B. Treat, 771 Broadway. 1887.

CONTRIBUTION TO THE STUDY OF THE HEART AND LUNGS. By JAMES R. LEAMING, M. D. New York: E. B. Treat, 771 Broadway. 1887.

These two volumes form the fifth and sixth numbers of Treat's "Medical Classics." Dr. Clark was for many years known as, in many respects, the most accomplished teacher of medical practice in America. As a lecturer he was direct, forcible, clear and logical. To such a complete system had he reduced his teachings that there was little difficulty in following him and comprehending all he said. He was eminently "instructive," and that is a leading characteristic of his book. All the charms of his didactic lectures seem to be preserved in its pages, and even when one opens it at random and commences a kind of aimless perusal, he is certain soon to have his interest aroused and to follow the chapter to the end. Then a moment's reflection will probably surprise him at the amount of information which has been given. Furthermore, it has been presented in such an attractive manner, and the subject has been made so clear and lucid, that the points made will not soon be forgotten. In fact, the book reads like a novel, and the subject matter is as easily followed as a biography. It is admirably adapted for "pick-up" use—for reading at odd minutes—because its every sentence is so clear that it will stand alone.

The work of Dr. Leaming is more general in its scope, inasmuch as it covers, not only diseases of the heart, but of the respiratory organs and others which are relative to them. It is a collection of monographs, which first appeared as contributions to medical journals and papers read before medical societies. These are now systematized, connected and published in book form. Prof. Leaming is known as a very acute observer in pathological conditions. He has a natural quickness of ear and perception, and these have been cultivated by many years of practice almost to the point of perfection. As a consequence, his judgment in questions of diagnosis is always remarkably clear, and he is enabled to point out to the student many facts and symptoms which a less careful observer would miss altogether. In the consideration of the studies of the therapeutics of chloride of ammonium and of mercury, for instance, the author has given the most minute attention to the effects produced by their exhibition, and from these observations, which are recorded with great particularity, he is enabled to deduce many useful lessons. The book is undoubtedly a valuable contribution to our knowledge of chest diseases.

The Student's Manual and Hand-Book for the Dental Laboratory. By L. P. Haskell. Philadelphia: The Welch Dental Company. 1887.

Dr. Haskell is too well known in prosthetic dentistry to need any introduction at our hands—or, indeed, those of any one else. Possessed of intuitive mechanical intelligence, and with a life-time of solid experience back of him, his suggestions to the dental student are worth attention. He is one of the "old-timers" who did not learn his profession in the age of the "cheap and nasty." In the student days of Dr. Haskell dentists were not made in three months, and there were no diploma mills to grind out a batch on the shortest possible notice. At that time a knowledge of metallurgy was a necessity, and the dentist who was not quite competent to refine his own gold, roll it out and work it into any conceivable shape, could not get a situation as a sweeper and cleaner. Many of his hints are thrown away upon students of the Baconian school, whose fit-out consists of a pair of forceps, a lathe, a vulcanizer and two or three scrapers. But he who proposes to work a laboratory for what it is worth will find much that is valuable. To be sure, we cannot always agree with him, and some of his methods we long ago discarded, while he condemns some of our pet notions, but these are usually the results of habit, and Dr. Haskell's mode of working will commend itself to very many.

Ueber Bissarten und Bissanomalien. Ein Studie von Dr. Alfred Sternfeld, Approb. Arzt in München.

(Different Forms of Bite and Bite Anomalies. A study by Dr. Alfred Sternfeld; a Qualified Physician in Munich.)

This is a work that deserves more attention than can be given it in a brief review. The author says that as he finds the term "bite"—occlusion of the teeth—practically unknown in medical literature, he feels called upon to define it, which he does in a general way, as the manner in which the lower teeth meet the upper; in a special sense, the position assumed by a toothless maxilla toward one that has teeth. In this view he proceeds to describe all forms of normal bite, and how they can be influenced artificially. The second half treats of abnormal bites in the same systematic manner. An index and a brief resumé of the literature of the subject makes this useful work yet more complete.

The author criticises dental nomenclature, and sometimes very justly. For instance, while, as he says, the terms "distal" and "mesial" are accepted in dentistry as descriptive of the sides of the teeth, they are in that meaning unscientific, and should properly be applied to the cutting or grinding surface, and the root-ends. The work is profusely illustrated, but we cannot say much for the artistic value of the cuts.

DAS FÜLLEN DER ZÄHNE BEI INTACTER PULPA. VOL LUDWIG WARNEKROS, ZAHNARZT IN BERLIN. Verlag von C. Ash & Sons. 1888.

(The Filling of Teeth with Intact Pulps. By L. Warnekros, Dentist in Berlin.)

This very handsomely printed work is dedicated to the Dental Department of the University of Berlin. After an introductory chapter on Caries, the different methods of filling teeth, the keeping of cavities dry, etc., the author in consecutive chapters considers the different forms of cavities, and the best materials and methods for filling them. The volume is handsomely illustrated, and forms a valuable addition to the libraries of those who read the German language.

PHOTOGRAPHIC ILLUSTRATIONS OF SKIN DISEASES. A complete work on Dermatology. An Atlas and Text-Book Combined. By GEORGE HENRY FOX, A. M., M. D. New York: E. B. Treat, 771 Broadway.

Parts Three and Four of this invaluable and beautiful work contain illustrations of Eczema (rubrum et squamosum, squamosum 3, intertrigo, faciei 2, barbae and syphiloderma) Sycosis, Trichiphytosis, Dermatitis and Psoriasis. Some of the hand-colored plates are wonderfully true representations of these diseases, and the series will be simply essential to every dermatologist.

(A considerable number of pamphlets and small publications have reached us, but it is absolutely impossible that we should find room to notice them in this number.)

